# First person – Christoph Bruns

**DOI:** 10.1242/bio.062153

**Published:** 2025-07-31

## Abstract

First Person is a series of interviews with the first authors of a selection of papers published in Biology Open, helping researchers promote themselves alongside their papers. Christoph Bruns is first author on ‘ Cuticular microstructure of the locust femur-tibia joint’, published in BiO. Christoph is a PhD student in the lab of Dr Jan-Henning Dirks at Hochschule Bremen City University of Applied Sciences, Bremen, Germany, investigating the mechanical properties and functional morphology of insect cuticle, with a particular focus on fatigue-induced microdamage and fracture behavior.


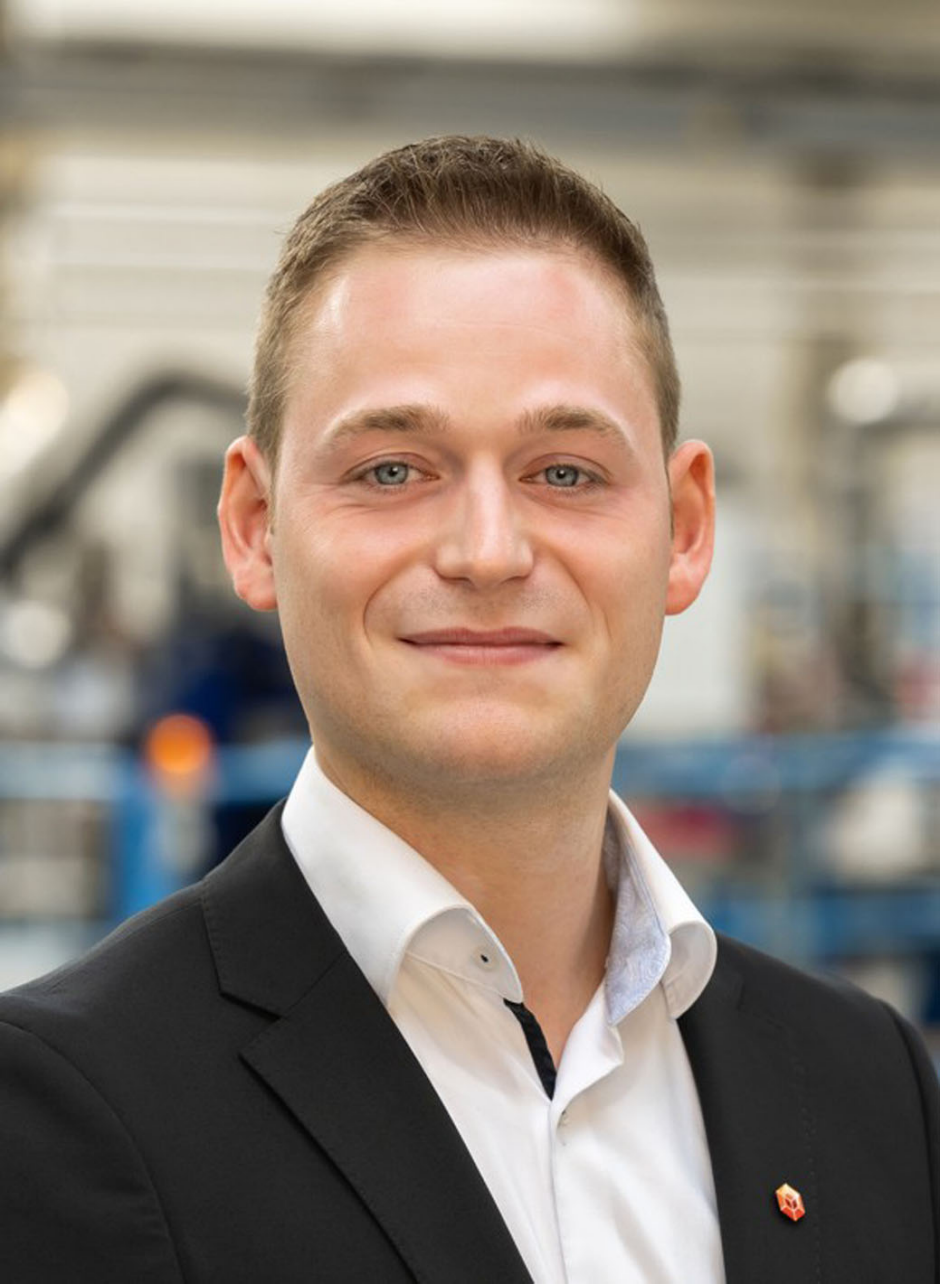


**Christoph Bruns**

**Describe your scientific journey and your current research focus**

I think my scientific journey, especially into the field I'm working in now, has been rather unusual. I come from a technical background, with a bachelor's in materials science focused on lightweight structures and a master's in biomimetics. After working in industry as a validation engineer, testing prototypes for an automotive supplier, I returned to academia to join a research project on bioinspired orthoses where I was also planning my PhD project. When that project ended and I was still waiting for funding approval of my PhD-project, I temporarily switched fields again and worked at the Leibniz Institute for Materials Engineering in Bremen. It wasn't a straight path, but these detours helped me gain experience and confirm my passion for research. I'm now in my second year of a PhD at the City University of Applied Sciences in Bremen, investigating the mechanical properties and fatigue behavior of insect cuticle, an exciting interdisciplinary project where I try to apply engineering standards to the mechanical testing of biological samples.

It wasn't a straight path, but these detours helped me gain experience and confirm my passion for research.

**Who or what inspired you to become a scientist?**

I've always been curious, even as a child I was fascinated by how things work. Science programs and non-fiction books for children definitely awakened this early interest. Later, during my studies, some professors had a big influence, especially Professor Dr Susanna Labisch and Professor Dr Jan-Henning Dirks. They encouraged me to consider an academic career after my graduation and told me that my master's thesis showed that I was suited to academic work. This kind of personal encouragement is certainly of enormous importance in attracting young people into science in the future. And of course, I probably wouldn't have chosen this path if my family hadn't confirmed and supported me in my plans.

**How would you explain the main finding of your paper?**

We know surprisingly little of how insect joints are built and how they actually function, although this is an evolutionarily extremely successful mechanism. In my research, I had a closer look at the joints of locust legs and found that the membranes connecting the hard leg segments of the exoskeleton have a very characteristic folding. This means that the membranes do not stretch during movement but are primarily folded in a controlled manner to enable movement with as minimal resistance as possible. Some of these folds are related to internal structures like muscles, while smaller folds are likely influenced by the material properties and thickness of the membrane or its surface structure.

**What are the potential implications of this finding for your field of research?**

First, this helps us to better understand the organism itself, i.e. how insect joints are built and function. Of course, the findings could also inspire us to come up with new ideas for technical joint systems or soft robotics, for example.

**Figure:**
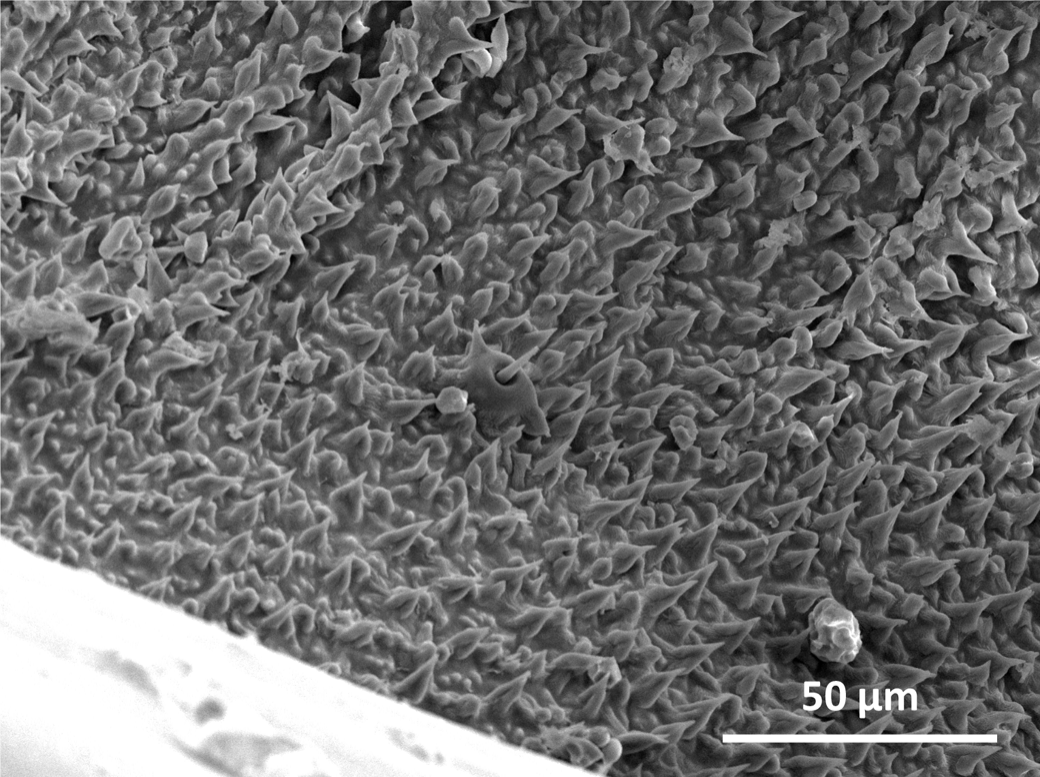
**SEM image of the membrane surface in the femur-tibia joint of *Locusta migratoria*.** The image shows the membrane region that is densely covered with cuticular spines.

**What do you enjoy most about being an early career researcher?**

What I enjoy most is that, especially as an early career researcher, you're constantly investing in yourself. You gain a huge amount of knowledge and develop a wide range of new skills in a very short time. I've worked in both industry and academia, and I've really enjoyed both, but the learning experience is quite different. In industry, responsibilities are often clearly divided, and if something falls outside your role, it's usually passed on to someone specialized in that task, even if you're interested in learning it yourself. That makes sense from an efficiency point of view, of course. In research, especially during a PhD, it's different: you need to understand every aspect of your project in detail, and that means teaching yourself new methods, solving problems independently, and sometimes going through trial and error. It can be frustrating at times, but that's exactly what makes it so rewarding. Nowhere else do you grow so quickly, both personally and professionally.

**What piece of advice would you give to the next generation of researchers?**

I never really planned to get to where I am now; but I always enjoyed what I was doing and so one thing led to another. I would advise you to follow what really interests you and not make every decision based on what seems best for your career on paper. I'm not sure if that's the smartest or most strategic path in science, but it's the one I've chosen - and so far it's kept me motivated and curious, which I think is essential for good research.

**What's next for you?**

To be honest, I have no idea yet. At the moment I'm fully focused on my PhD and that will definitely be my priority this year. When I enter my third year, I will start exploring possible career paths and opportunities for what comes after.

To maintain public trust in science, we need to bring science to the people

**Where could science improve?**

I think one of the most important challenges is to make science and especially fundamental research more visible and understandable to the general public. I live in a small village where most people don't have much contact with science. One of the most common questions I get when I talk about my research is: ‘What can you actually do with it?’ People outside of science often expect science to produce ready-to-use products, but it doesn't work like that. We need to make people understand that science is about generating knowledge, and that this knowledge forms the basis for future innovations. Many challenges scientists face, such as the lack of a clear perspective, temporary contracts or difficulties in funding research, are well known within the academia bubble but often invisible outside of it. When I talk to people in my community about this, they're usually surprised and strongly supportive of change. To maintain public trust in science, we need to bring science to the people. That means not only giving talks at conferences, but also speaking to audiences who rarely encounter scientific work. I've done this with three other scientists by organizing public talks in an old barn in our village and the feedback was wonderful and has really made people think.
